# Macrophages mediate corticotomy-accelerated orthodontic tooth movement

**DOI:** 10.1038/s41598-018-34907-5

**Published:** 2018-11-14

**Authors:** Yan Wang, Hanwen Zhang, Wen Sun, Siyu Wang, Shuting Zhang, Linlin Zhu, Yali Chen, Lizhe Xie, Zongyang Sun, Bin Yan

**Affiliations:** 1 Jiangsu Key Laboratory of Oral Diseases, Nanjing Medical University, Nanjing, China; 2 Department of Orthodontics, Affiliated Hospital of Stomatology, Nanjing Medical University, Nanjing, China; 3 School of Basic Medical Sciences, Nanjing Medical University, Nanjing, China; 4 Division of Orthodontics, College of Dentistry, Ohio State University, Columbus, USA; 5 Suzhou Hospital Affiliated to Nanjing Medical University, Suzhou Science & Technology Town Hospital, 215153 Suzhou, Jiangsu Province China

## Abstract

Clinical evidence has suggested that surgical corticotomy of the alveolar bone can accelerate local orthodontic tooth movement (OTM), but the underlying cell and molecular mechanisms remain largely unclear. The present study examined the role of macrophages played in corticotomy-assisted OTM. Orthodontic nickel-titanium springs were applied to the left maxillary first molars of rats or mice to induce OTM with or without corticotomy. Corticotomy enhanced OTM distance by accelerating movement through induction of local osteoclastogenesis and macrophage infiltration during OTM. Further analysis showed that macrophages were polarized toward an M1-like phenotype immediately after corticotomy and then switched to an M2-like phenotype during OTM. The microenvironment of corticotomy induced macrophage infiltration and polarization through the production of TNF-α. More importantly, the amount of OTM induced by corticotomy was significantly decreased after mice were depleted of monocyte/macrophages by injection of liposome-encapsulated clodronate. Further experiments by incubating cultured macrophages with fresh tissue suspension obtained from post-corticotomy gingiva switched the cells to an M1 phenotype through activation of the nuclear factor-κB (NF-κB) signaling pathway, and to an M2 phenotype through activation of the JAK/STAT3 signaling pathway. Our results suggest that corticotomy induces macrophage polarization first by activating the NF-κB signaling pathway and later by activating the JAK/STAT3 signaling pathway, and that these processes contribute to OTM by triggering production of inflammatory cytokines and osteoclastogenesis.

## Introduction

The average duration of a comprehensive orthodontic treatment is 20–36 months^1^. As prolonged treatment increases the risk of periodontal disease and root resorption, especially in adult patients^2,3^, orthodontic professionals are constantly seeking ways to accelerate orthodontic tooth movement (OTM)^4^. To date, the only approach that has been confirmed by high-quality randomized clinical trials to be effective in accelerating OTM is corticotomy of local alveolar bone^5–7^. The effectiveness of this approach has been largely attributed to a so-called regional acceleratory phenomenon (RAP)^8^. However, the specific cell and molecular players that are involved in the RAP response and are stimulatory for OTM remain largely unknown.

One potentially critical cell player for corticotomy-accelerated OTM is macrophage. Macrophages regulate tissue homeostasis in various pathophysiologic processes, including innate and adaptive immunity, wound healing, hematopoiesis, and malignancy^9^. A typical innate immune response following bone injury is characterized by an initial infiltration of polymorphonuclear neutrophils that secrete inflammatory and chemotactic mediators, followed by an influx of large quantities of monocytes and macrophages^10^. Broadly speaking, the main functions of macrophages are twofold: inflammation and regeneration, respectively, by M1 and M2-polarized subtype macrophages^9^. Both inflammatory and regenerative events have been found to be essential for wound healing in bone^11^ as well as mechanically-induced OTM^12^. In the latter process, compressive forces produce tissue destruction and bone resorption representing inflammation on one side of the tooth^13^, while tensile force leads to bone formation on the other side^14^.

Macrophages have been found to be centrally involved in changes on the compression side of OTM. A recent study found that polarization of M1-like macrophages was critical for alveolar bone resorption on the compression side and subsequent onset of OTM, and monocyte/macrophage depletion drastically reduced the amount of osteoclasts and the OTM distance^15^. These findings strongly suggest that corticotomy-accelerated OTM is mediated by local monocytes/macrophages. Whether this notion is true has not been tested in any experimental or clinical studies so far, and the role of macrophage polarization in orthodontic tooth movement, particularly in corticotomy-assisted tooth movement, is yet to be elucidated. The purpose of this study, therefore, was to investigate the role of macrophages played in corticotomy-accelerated OTM. We asked two specific questions: whether corticotomy alters the microenvironment of macrophages and affects their polarization and infiltration, and what molecular pathways are involved in these changes.

## Materials and Methods

### Animal models

Adult Wistar rats (male, 220–240 g, 8 weeks old) and C57BL/6 mice (male, 25–28 g, 8 weeks old) were used in this study. All animals were maintained in a virus- and parasite-free barrier facility and exposed to a 12-h/12-h light/dark cycle under standard conditions in the Medical Experimental Animal Center of Nanjing Medical University, China. All live animal procedures were approved by the Committee of Nanjing Medical University for Animal Resources (approval ID: IACUC-14030141 for rat and IACUC-1601118 for mouse).

### Application of orthodontic devices and corticotomy

Both the rats and mice were divided into tooth movement only (TM) or corticotomy-assisted tooth movement (CO + TM) groups. To induce tooth movement, the left maxillary first molars were ligated to the maxillary incisors using NiTi coil springs, with a force of approximately 60 g for rats (Appendix Fig. 1A) and 30 g for mice, which were chosen based on previous studies^15,16^. For the CO + TM groups, the corticotomy procedure was similar to that reported by others^17,18^. Briefly, two vertical corticotomies, 7 mm long and 1 mm deep, were made supraperiosteally to the mesial and distal side of the left maxillary first molar palatal alveolar process in rats using a #11 surgical blade (Appendix Fig. 1B,C). In mice, the corticotomies were the same except for being 3 mm long. For consistency, the corticotomies were conducted by the same investigator after practicing on multiple test animals. For each corticotomy, the surgical blade was inserted gradually through the palatal gingiva and into the cortical plate (Appendix Fig. 1B,C), using tactile sense to confirm the penetrations. All the operations conducted on animals were according to the protocols approved by the Committee of Nanjing Medical University for Animal Resources.

The force appliances were monitored every 24 h and reinstalled promptly if there were breakages. After appliance activation, the animals were fed softened standard rat chow to minimize discomfort and the risk of appliance damage or dislodgement. The rats in each group were sacrificed 3, 5, 7, 14, 21, 28, and 42 days after force application for tissue collection. The mice in each group were sacrificed 5, 7, 14, and 21 days after force application.

### *In vivo* depletion of monocytes/macrophages

Mice in both TM and CO + TM groups were randomly divided into two groups (n = 5–6 per group): one received intravenous injection of liposome-encapsulated clodronate (200 μL/mouse, 5 mg/mL; Liposoma B.V., Netherlands) to deplete monocytes/macrophages^19^; the other received intravenous injection of phosphate-buffered saline (PBS, control). All mice received injections every 4 days starting 1 day before force application until 21 days after force application (Appendix Fig. 1D). The number of CD11b^+^F4/80^+^ macrophages in the spleen and gingiva was analyzed using flow cytometry to assess the depletion effects. The contribution of liposome-encapsulated clodronate injection to macrophage polarization was further determined based on the numbers of CD86^+^ and CD206^+^ cells among CD11b^+^ F4/80^+^ cells in gingiva using flow cytometry.

### Tissue collection and preparation

Upon reaching the designed termination time, all animals were euthanized by carbon dioxide overdose. After euthanasia, the palatal gingiva of the left maxillary first molar was immediately collected for reverse transcription–polymerase chain reaction (qRT-PCR) and flow cytometry analyses. Then, the maxillae were fixed with 4% paraformaldehyde for 48 h, followed by paraffin embedding.

The collected palatal gingiva of 5 mice in each group was ground up in Dulbecco’s modified Eagle’s medium (DMEM; Gibco, CA, USA) containing 1% fetal bovine serum (FBS; ScienCell, CA, USA), chopped, and filtered through a nylon grid of 200 μm mesh. The solutions were filtered through a 0.22-μm filter (Millipore, MA, USA), and the protein concentration was determined using the Bicinchoninic Acid Protein Assay Kit (Thermo Fisher Scientific, IL, USA) according to the manufacturer’s instructions. Bovine serum albumin (BSA) was used as the standard.

### Isolation, culture and treatment of bone marrow–derived macrophages

A murine fibroblastic cell line, L929, was used as a source of M-CSF/CSF-1. The cells were cultured to confluence in DMEM with 10% FBS. Cell-free conditioned medium was harvested and stored under −80 °C until use.

Bone marrow–derived macrophages (BMDMs) were isolated and cultured as described^20^. Briefly, bone marrow cells from 2-month-old mice were flushed out from the bone marrow cavity of femurs and tibias. The cells were then washed, resuspended in DMEM containing 10% FBS, and seeded into tissue culture plates. After initial overnight incubation, non-adherent cells were removed by gentle pipette aspiration. Adherent cells were cultured for 7 days in DMEM supplemented with 10% FBS, 2 mM glutamine, antibiotics (100 U/mL of penicillin and 100 mg/mL of streptomycin; Gibco), and 25% (v/v) L929-conditioned medium. The cells were then cultured with the fresh tissue suspension of palatal gingiva tissues from mice with or without corticotomy for 24 h.

### Micro-computed tomography scanning and three-dimensional reconstruction

Six samples of maxillae were selected randomly from each group. All samples were scanned using a micro-computed tomography (CT) machine (Skyscan 1176, Bruker, Germany) with a standard acquisition protocol (50 kV, 500 μm, and 0.3 mm voxel size). A single-blinded rater renamed the micro-CT data files with randomly generated codes. The files were then imported into Dataviewer (Bruker) and reconstructed for analysis. The height of the alveolar bone crest at the distal bone ridge of the tooth was measured on two-dimensional sagittal sections taken through the centers of the upper left first and second molars. The distance was measured between the most mesial point of the second molar crown and the most distal point of the first molar crown; this parameter is also known as the distance between the first and second molar. In all animals, the convex crown surfaces were touching at the start of the study, corresponding to an initial separation distance of 0 mm before tooth movement^21^. The average movement rate was calculated as the amount of tooth movement divided by the number of days.

The reconstructed data were imported into CTan (Bruker) to detect the structure of the alveolar bone. The volume of interest (VOI) surrounding the left maxillary first molar was demarcated using the following boundaries: the mesial-most edge of the first molar, the mesial-most edge of the second molar, the lateral-most edge of buccal and palatal alveolar cortical plates, the apical edge of the first molar root tip, and the coronal-most edge of the first molar root furcation^22^. The bone mineral density (BMD) of the VOI was measured. All subjects were measured three times every week by the same rater, and the mean measurement was used as the BMD. Three-dimensional reconstruction of the maxillae was then performed using MIMICS 17.0 (Materialise, Leuven, Belgium). The main procedures were the segmentation of a series of sectional images and 3D rendering of the region of interest, i.e. the upper left molars and the alveolar bone.

### Tartrate-resistant acid phosphatase staining (TRAP)

The sections mesial and distal to the left maxillary molars of rats were fixed in 4% paraformaldehyde, demineralized in 15% ethylenediaminetetraacetic acid, and embedded in paraffin. All sections (5 μm thickness) were cut along the vertical meridian and deparaffinized in xylene, dehydrated in gradient ethanol, and then washed with PBS before staining using tartrate-resistant acid phosphatase (TRAP) or immunohistochemistry.

For TRAP staining, dewaxed sections were preincubated for 20 min in a buffer containing 50 mM sodium acetate and 40 mM sodium tartrate (pH 5.0). Sections were incubated for 15 min at room temperature in the same buffer containing 2.5 mg/mL naphthol AS-MX phosphate in dimethylformamide as substrate and 0.5 mg/mL Fast Garnet GBC (Sigma-Aldrich) as color indicator for the reaction product. Sections were washed with distilled water, counterstained with methyl green and mounted in Kaiser’s glycerol jelly. Osteoclasts were identified as multinucleated, TRAP-positive cells in contact with or near bone surfaces and in the periodontal ligament.

### Immunohistochemistry

For immunohistochemistry, sections were dipped in 10 mM citrate buffer (pH 6.0) and heated in a microwave oven for 15 min to thermally induce antigen epitope retrieval. After pretreatment with 3% H_2_O_2_ in PBS for 15 min to inactivate endogenous peroxidase activity and blocking with 5% BSA at room temperature, the sections were incubated with antibodies (Abcam, MA, USA) against CD68, CD11b, CD86, or CD163. Sections were washed extensively with PBS, incubated with relevant secondary antibodies, washed again, stained with 3,3′-diaminobenzidine (Dako, CA, USA), and finally counterstained with hematoxylin (Boster, Wuhan, China).

All images were captured from five consecutive microscopic fields using an Olympus BX40 light microscope (Olympus, Japan) coupled to a Dinolite AM423X microcamera (AmMo Electronics Corp, Taiwan) at 200 × magnification and processed with Image-Pro Plus 6.0 software.

### RNA preparation and quantitative real-time polymerase chain reaction (qRT-PCR)

Total RNA was isolated from tissues or macrophages after various treatments using TRIzol reagent (Thermo Fisher Scientific, USA) according to the manufacturer’s instructions. Complementary DNA was amplified from 2.0 μg of total RNA in a final volume of 10 μL using PrimeScript Reverse Transcription Master Mix (TaKaRa, Dalian, China), then quantitative real-time PCR was performed using SYBR Green Premix Ex Taq (TaKaRa) on an ABI 7900 (Applied Biosystems, USA) using the following parameters: 95 °C for 10 min, followed by 40 cycles of 95 °C for 15 s and 60 °C for 1 min. Data for all genes were processed using the 2^−ΔΔCt^ method and normalized to the internal control glyceraldehyde-3-phosphate dehydrogenase (GAPDH). The primers used for real-time PCR are listed in Appendix Table 1.

### Flow cytometry

The gingiva tissue was washed with ice-cold 1% FBS in PBS, then chopped and filtered through a nylon grid of 200 μm mesh. Macrophages were isolated using gradient centrifugation, then incubated on ice for 30 min using fluorescently conjugated antibodies of the following, rat CD11b (APC-conjugated; BD Biosciences, NJ, USA), rat CD86 [fluorescein isothiocyanate (FITC)-conjugated; AbDSerotec, CA, USA], rat CD163 (FITC; AbDSerotec), mouse F4/80 (PerCP-Cyanine5.5-conjugated; eBioscience, CA, USA), mouse CD11b (APC-conjugated; eBioscience), mouse CD86 (FITC-conjugated; eBioscience), and mouse CD206 (PE-conjugated; eBioscience). The percentages of positively stained cells were analyzed using a BD FACSCalibur flow cytometer (BD Biosciences) with Cell Quest Pro software (BD Biosciences). Isotype controls were used to set appropriate gates. For all samples, approximately 10,000 cells were analyzed for at least three times.

### Cell lysis and Western blotting

Western blotting was performed according to standard procedures. Briefly, macrophages treated as in different ways were lysed with radioimmunoprecipitation assay (RIPA) buffer (Santa Cruz, CA, USA), total proteins were extracted, and the proteins were separated on 10% sodium dodecyl sulfate–polyacrylamide gels. Proteins were then transferred to polyvinylidenedifluoride membranes (Millipore, MA, USA), which were blocked with 5% skim milk, and incubated overnight with primary antibodies against p65 (Proteintech, Wuhan, China); STAT3 or phospho-STAT3 (Cell Signaling Technology, MA, USA); or GAPDH (Kangcheng Biological, Shanghai, China). Membranes were then incubated for 1 h with horseradish peroxidase–conjugated secondary antibodies (Shengxing Biological, Nanjing, China), and exposed using enhanced chemiluminescence (Millipore, MA, USA). Expression levels were quantified using Image Lab software (Bio-Rad, CA, USA) and normalized to GAPDH.

### Statistical analysis

Statistical analysis was performed using SPSS 19.0. Mean and standard deviation values were based on the sample size or triplicates of *in vitro* experiments. Two group-based comparisons were analyzed by two-tailed Student’s *t* tests. Three or more group-based comparisons were conducted using one-way analysis of variance followed by Dunnett’s post hoc multiple comparisons. A significance level of less than 0.05 was adopted.

## Results

### Corticotomy increased the rate of OTM

**Figure 1:**
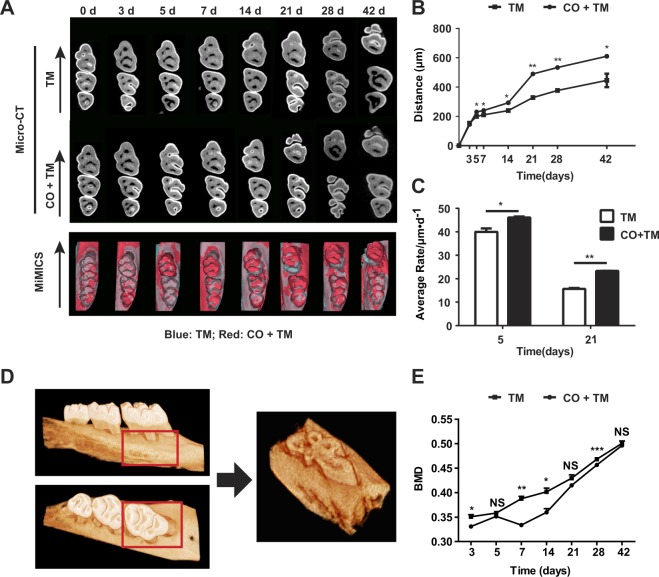
Corticotomy increased the rate of OTM. Rats (male, 220–240 g, 8 wk old) (**A**–**E**) were divided into tooth movement only (TM) or corticotomy-assisted tooth movement (CO + TM). The rats (**A**–**E**) in each group were sacrificed at 3, 5, 7, 14, 21, 28 and 42 days for tissue collection and assessments. (**A**) Representative micro–computed tomography images and combined three-dimensional models established by mimics in rats from day 0 to day 42. Arrows represent the direction of force application. N = 5 or 6. Blue: TM; Red: CO + TM. (**B**) Semiquantification of orthodontic tooth movement (OTM) distance in rats from day 0 to day 42 (n = 5 or 6). (**C**) Average rate of orthodontic tooth movement (OTM) in rats on days 5 and 21 (n = 5 or 6). (**D**) The volume of interest (VOI) surrounding the left maxillary first molar was demarcated using the following boundaries: the mesial-most edge of the first molar, the mesial-most edge of the second molar, the lateral-most edge of buccal and palatal alveolar cortical plates, the apical edge of the first molar root tip, and the coronal-most edge of the first molar root furcation. (**E**) The bone mineral density (BMD) of the VOI was measured to assess the mineralization that occurred during tooth movement with or without corticotomy. Micro-computed tomography was performed from day 3 to day 42 after force was applied. *p < 0.05, **p < 0.01, ***p < 0.001 *vs* TM group. NS, not significant.

OTM in the TM and CO + TM groups of rats was examined by micro-CT scans. Data from sequentially sacrificed groups of rats showed progressive and substantial tooth movement in both groups (Fig. 1A,B). The amount of accumulative tooth movement was significantly greater in the CO + TM group from day 5 to day 42 than that in the TM group at each time point. Comparison of average OTM rate showed that the CO + TM group was significantly higher on days 5 and 21 than TM only (Fig. 1C).

Bone mineral density (BMD) of the volume of interest (VOI) was measured from micro-CT scans (Fig. 1D,E). It increased with time in both groups and was significantly lower in the CO + TM group than in the TM group at multiple time points (days 3, 7, 14 and 28) (Fig. 1E). Furthermore, we measured the distal side alveolar bone height of the upper left first molar, which showed a little more bone height reduction in the CO + TM group than the TM group on day 42 (Appendix Fig. 2).

### Corticotomy induced osteoclastogenesis and macrophage infiltration during OTM

**Figure 2:**
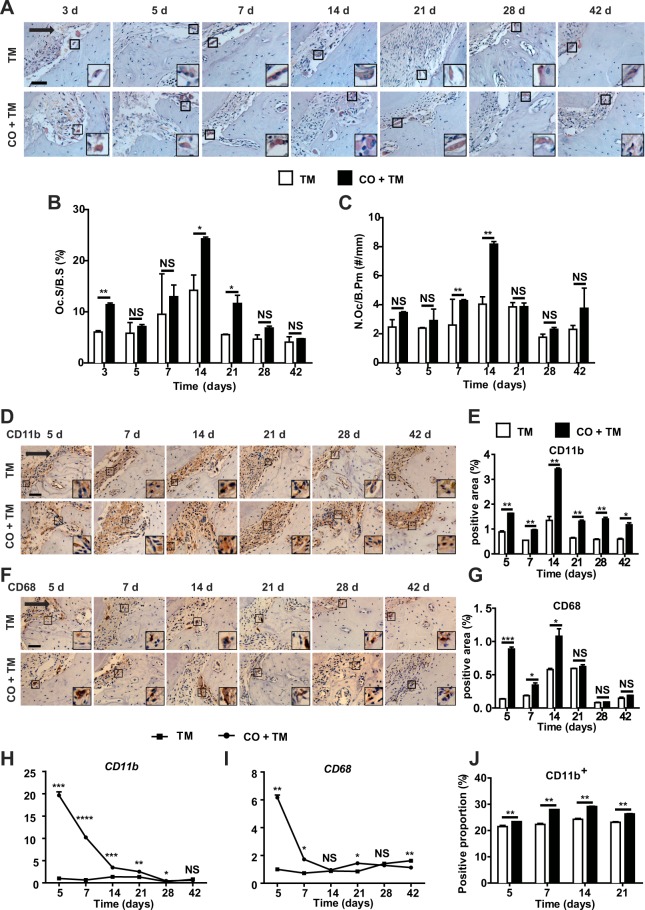
Corticotomy induced osteoclastogenesis and macrophage infiltration during OTM. Rats (male, 220–240 g, 8 wk old) (**A**–**J**) were divided into tooth movement only (TM) or corticotomy-assisted tooth movement (CO + TM). The rats (**A**–**I**) in each group were sacrificed at 3, 5, 7, 14, 21, 28 and 42 days or specified in each group for tissue collection and assessments. (**A**) Representative tartrate-resistant acid phosphatase (TRAP) staining of the compression side of the distobuccal roots. Large boxed areas show high-magnification views of the small boxed areas. The arrow represents the direction of force application. Scale bar: 50 μm. The surface of osteoclasts relative to the bone surface (Oc.S/B.S, %) (**B**) and the number of osteoclasts per mm of bone parameter (N.Oc/B.Pm, #/mm) (**C**) were determined in the dental alveolar bone of TRAP-stained mandibles. Data are the mean ± SD from five fields. (**D**,**F**) Representative images of the compression side of distobuccal roots immunostained against CD11b (**D**) or CD68 (**F**) after force was applied for 5, 7, 14, 21, 28 or 42 days. Large boxed areas show high-magnification views of the small boxed areas. The arrow represents the direction of force application. Scale bar: 50 μm. CD11b-positive areas (**E**) and CD68-positive areas (**G**) were calculated as a percentage of the total tissue area on the compression side for each subject. Real-time PCR analysis of CD11b (**H**) and CD68 (**I**) expression in gingiva after force was applied for 5 to 42 days with or without corticotomy surgery. Data for each gene were processed using the 2^−ΔΔCt^ method and expressed relative to GAPDH. Data are mean ± SD of at least three independent experiments. (**J**) Flow cytometry analysis of the proportions of CD11b-positive cells in gingiva of rats after force was applied for 5 to 21 days with or without corticotomy surgery. Data are mean ± SD from three independent experiments. *p < 0.05, **p < 0.01, ***p < 0.001, ****p < 0.0001 *vs* TM group. NS, not significant.

Osteoclast activity was evaluated by immunohistochemical staining for tartrate-resistant acid activity (TRAP). Overall, the density of TRAP-positive cells was greater in the CO + TM group than in the TM group, although the difference only reached significance at day 3, 14 and 21 (Fig. 2A–C). Consistent between the two parameters (Oc.S/B.S and N.Oc/B. Pm), osteoclast density peaked at day 14 and was significantly higher in the CO + TM group than the TM group.

Macrophages on the compression side were stained with antibodies against CD11b (Fig. 2D) and CD68 (Fig. 2F), commonly used macrophage markers related to lysosomal glycoproteins. Immunohistochemistry showed significantly greater positive area for CD11b^+^ macrophages in the CO + TM group than in the TM group from day 5 to day 42 (Fig. 2D,E). The positive area for CD68^+^ macrophages was significantly higher in the CO + TM group than the TM group from day 5 to day 14 (Fig. 2F,G). Consistent with protein expression, real-time PCR results showed significantly higher relative expression of CD11b and CD68 mRNA in the CO + TM group than in the TM group from day 5 to day 21 (Fig. 2H,I). These findings were further verified using flow cytometry: the proportion of CD11b^+^ cells was significantly greater in the CO + TM group than in the TM group from day 5 to day 21 (Fig. 2J).

### Corticotomy induced macrophage polarization at different stages of OTM

**Figure 3:**
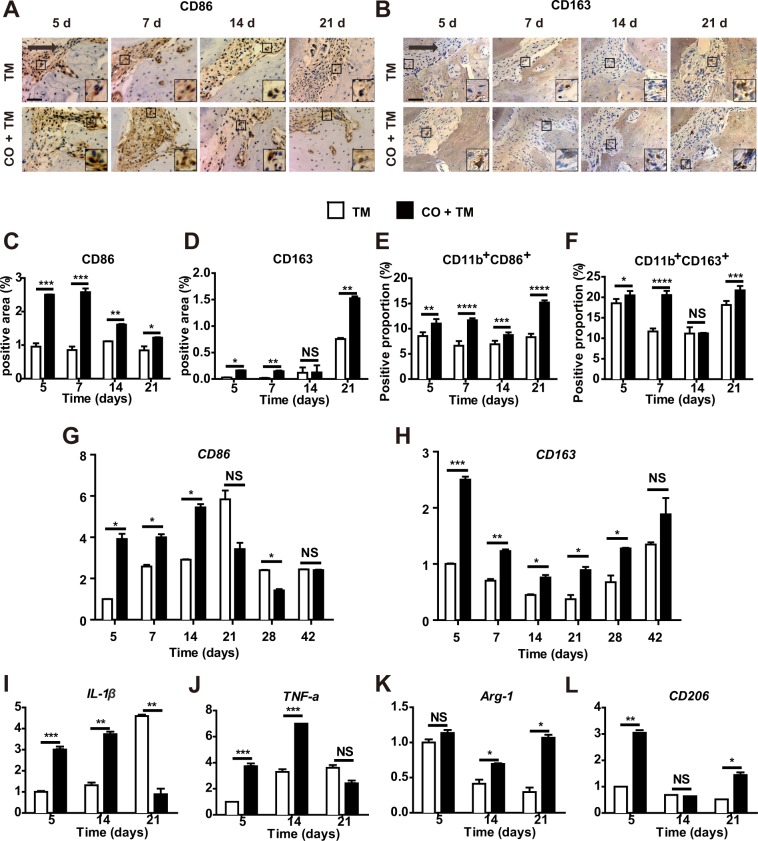
Corticotomy induced macrophage polarization at different stages of OTM. Rats (male, 220–240 g, 8 wk old) (**A–G**) were divided into tooth movement only (TM) or corticotomy-assisted tooth movement (CO + TM). The rats (**A–L**) in each group were sacrificed at 5, 7, 14, 21, 28 and 42 days or specified in each group for tissue collection and assessments. (**A,B**) Representative images on the compression side of distobuccal roots immunostained against CD86 (**A**) or CD163 (**B**) after force was applied for 5, 7, 14 or 21 days. Large boxed areas show high-magnification views of the small boxed areas. The arrow represents the direction of force application. Scale bar: 50 μm. CD86-positive areas (**C**) and CD163-positive areas (**D**) were calculated as a percentage of the total tissue area on the compression side. Flow cytometry analysis of proportions of CD11b^+^CD86^+^ cells (**E**) and CD11b^+^CD163^+^ cells (**F**) in gingiva of rats after force was applied for 5 to 21 days with or without corticotomy surgery. Data are mean ± SD of three independent experiments. Real-time PCR analysis of CD86 (**G**) and CD163 (**H**) expression levels in gingiva tissues of rats after force was applied for 5 to 42 days with or without corticotomy surgery. Real-time PCR analysis of expression levels of the M1 phenotype-related genes IL-1β (**I**) and TNF-α (**J**), as well as levels of the M2 phenotype-related genes Arg-1 (**K**) and CD206 (**L**) in gingiva tissues of rats after force was applied for 5, 14 and 21 days with or without corticotomy surgery. Data for each gene were processed using the 2^−ΔΔCt^ method and expressed relative to GAPDH. Data are mean ± SD of at least three independent experiments. *p < 0.05, **p < 0.01, ***p < 0.001, ****p < 0.0001 *vs* TM group. NS, not significant.

Since corticotomy triggers macrophage infiltration, we subsequently examined whether corticotomy also affects macrophage polarization. Immunostaining against the M1-phenotype marker CD86 and the M2-phenotype marker CD163 (Fig. 3A,B) revealed a significantly larger positive area for CD86^+^ macrophages on the compression side in the CO + TM group than in the TM group from day 5 to day 21 (Fig. 3C). The positive area for CD163^+^ macrophages was significantly greater in the CO + TM group than in the TM group on days 5, 7 and 21 (Fig. 3D). Flow cytometry analysis revealed a greater proportion of CD11b^+^CD86^+^ cells in the CO + TM group gingiva than in the TM group gingiva from day 5 to day 21 (Fig. 3E), and a greater proportion of CD11b^+^CD163^+^ cells in the CO + TM group than in the TM group on days 5, 7, and 21 (Fig. 3F). In the CO + TM group, the proportions of CD11b^+^CD86^+^ cells (Fig. 3E) and CD11b^+^CD163^+^ cells (Fig. 3F) kept increasing until day 7, then declined slightly before increasing dramatically on day 21 (Fig. 3E,F). The proportion of CD11b^+^CD163^+^ cells increased and peaked on day 5 in the TM group, whereas it peaked on day 7 in the CO + TM group. The proportion declined sharply on day 14, before increasing again on day 21 in both groups (Fig. 3F).

These results were confirmed at the mRNA level using real-time PCR. Relative expression of the CD86 mRNA increased until day 14 in the CO + TM group, then declined slightly (Fig. 3G). In contrast, CD163 mRNA levels increased on day 5 and then fell until day 21 when it increased again in both groups (Fig. 3H). Levels of CD86 mRNA were higher in the CO + TM group than in the TM group during initial and middle periods of treatment (between day 5 and day 14; Fig. 3G), whereas expression of CD163 was higher in the CO + TM group than in the TM group during the OTM (between days 5 and 42) (Fig. 3H). Nevertheless, the difference between groups was not statistically significant on day 42. CD86 up-regulation correlated with high expression of IL-1β and TNF-α on day 5 and day 14 in the CO + TM group (Fig. 3I,J). Expression of the M2-phenotype markers Arg-1 and CD206 was significantly higher in the CO + TM group than in the TM group on day 5 and day 21 (Fig. 3K,L).

### Corticotomy-induced OTM was decreased by macrophage depletion

**Figure 4:**
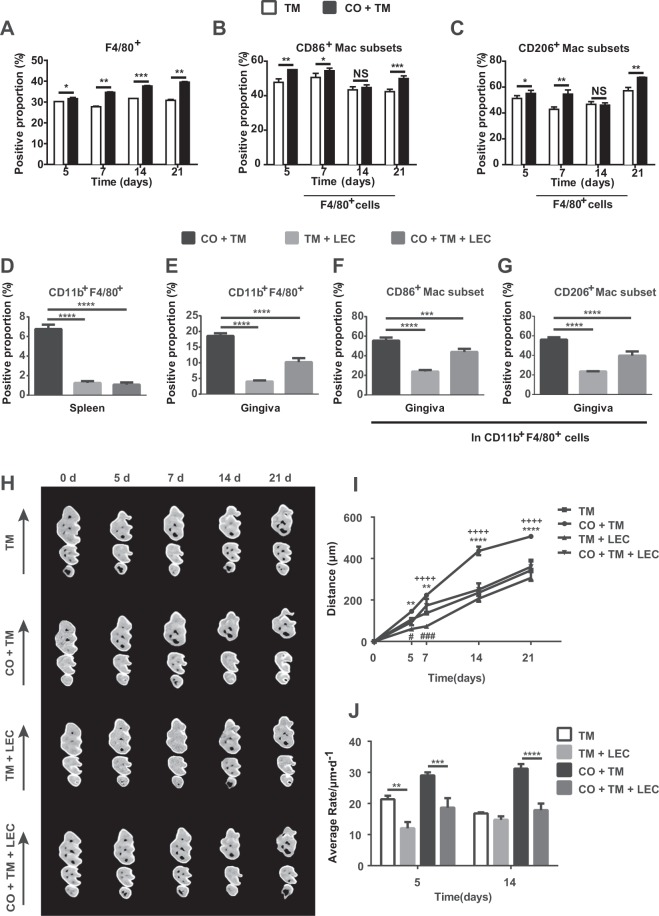
Corticotomy-induced OTM was decreased by macrophage depletion. Mice (male, 25–28 g, 8 wk old) (**A–C**) were divided into tooth movement only (TM) or corticotomy-assisted tooth movement (CO + TM). The mice (**A–J**) were sacrificed at 5, 7, 14 and 21 days. (**A**) Flow cytometry analysis of the proportions of F4/80-positive cells in gingiva of mice after force was applied for 5 to 21 days with or without corticotomy surgery. (**B,C**) Flow cytometry analysis of proportions of CD86-positive cells (**B**) and CD206-positive cells (**C**) in the F4/80-positive population in gingiva of mice after force was applied for 5 to 21 days with or without corticotomy surgery. Data are mean ± SD of three independent experiments. *p < 0.05, **p < 0.01, ***p < 0.001 *vs* TM group. NS, not significant. Both groups of TM and CO + TM mice were also injected intravenously with liposome-encapsulated clodronate (200 μL/mouse); these animals were labeled as the TM + LEC group and CO + TM + LEC group (**D**–**J**). Animals in the control group were intravenously injected with phosphate-buffered saline (200 μL/mouse) every 4 days starting 1 day before force application until 21 days after force application. Flow cytometry analysis of proportions of CD11b- and F4/80-positive cells in spleen (**D**) and gingiva (**E**) of mice after clodronate injection. (**F,G**) Flow cytometry analysis of proportions of CD86-positive cells (**F**) and CD206-positive cells (**G**) in the CD11b- and F4/80-positive population in gingiva of mice after clodronate injection. Data are mean ± SD of three independent experiments. ***p < 0.001, ****p < 0.0001 as indicated groups. Representative micro–computed tomography images (**H**) and semiquantification of OTM distance (**I**) in mice from day 0 to day 21 (n = 5 or 6). Arrows represent the direction of force application. ^**++++**^p < 0.0001 as CO + TM *vs* TM group. ^**#**^p < 0.05, ^**###**^p < 0.001 as TM + LEC *vs* TM group. **p < 0.01, ****p < 0.0001 as CO + TM + LEC *vs* CO + TM group. (**J**) Average rate of orthodontic tooth movement (OTM) in mice on days 5 and 14 (n = 5 or 6). **p < 0.01, ***p < 0.001, ****p < 0.0001 as indicated groups.

To further confirm the importance of macrophage infiltration in corticotomy-induced OTM, we examined the effect of macrophage depletion using a mouse OTM model. First, without macrophage depletion, the proportion of F4/80^+^ expression in the gingiva was significantly higher in the CO + TM group than in the TM group from day 5 to day 21 (Fig. 4A). Flow cytometry analysis further found that in the CO + TM group, the proportions of CD86^+^ and CD206^+^ cells among F4/80^+^ cells were significantly higher than those in the TM group on days 5, 7, and 21 (Fig. 4B,C). Next, after depleting macrophage by injecting liposome-encapsulated clodronate (LEC), which was confirmed by a 70% decrease in circulating monocytes (Fig. 4D), we found that the number of CD11b^+^F4/80^+^ macrophages in the gingiva of TM + LEC and CO + TM + LEC groups also significantly decreased (Fig. 4E). Flow cytometry analysis further demonstrated that macrophage polarization was affected by LEC injection, showing that the upregulation of both CD86^+^ and CD206^+^ cells among CD11b^+^F4/80^+^ cells induced by corticotomy was significantly repressed (Fig. 4F,G).

More importantly, without macrophage depletion, significantly larger tooth movement was shown in the CO + TM group than in the TM group from day 7 to day 21 (Fig. 4H,I). The amount of OTM was significantly decreased on days 5 and 7 in TM + LEC group than in the TM group with macrophage depletion, while the amount of OTM induced by corticotomy was also significantly decreased in the CO + TM + LEC group from day 5 to day 21 (Fig. 4H,I). The average rate of tooth movement was significantly lower in mice receiving LEC injection than those receiving saline injection on day 5, while the average rate of tooth movement in the CO + TM + LEC group was also significantly lower than in the CO + TM group on day 14 (Fig. 4J).

### Corticotomy induced macrophage polarization by activating NF-κB and JAK–STAT pathways

**Figure 5:**
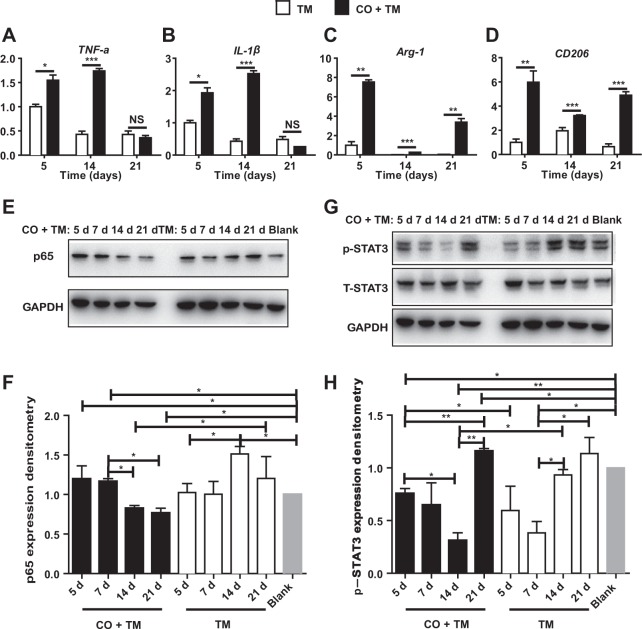
Corticotomy induced macrophage polarization by activating NF-κB and JAK–STAT pathways. Bone marrow cells (BMCs) from 2-month-old mice were cultured for 7 days with conditioned medium from L929 cells (L929-CM) to generate bone marrow–derived macrophages (BMDMs). Mice (male, 25–28 g, 8 wk old) (**A**–**H**) were divided into tooth movement only (TM), corticotomy-assisted tooth movement (CO + TM) and blank with neither tooth movement nor corticotomy. Tissue suspension was prepared from fresh palatal gingiva after corticotomy had been applied for 5 to 21 days as described in Materials and Methods. Real-time PCR analysis of the expression of genes encoding TNF-α (**A**), IL-1β (**B**), Arg-1 (**C**) or CD206 (**D**) in BMDMs after 24-h incubation with fresh tissue suspension of palatal gingiva tissues. Data for each gene were processed using the 2^−ΔΔCt^ method and expressed relative to GAPDH. Data are mean ± SD of at least three independent experiments. After incubation with fresh tissue suspension of palatal gingiva tissues, BMDMs were harvested and protein extracts were analyzed by Western blotting against p65 (**E**,**F**), phosphorylated STAT-3 (p-STAT3) and total STAT3 (T-STAT3) (**G**,**H**). Protein levels were calculated as a ratio to GAPDH and expressed relative to levels in the blank control. TM: tooth movement; CO + TM: tooth movement surgically assisted by corticotomy; Blank: blank group with no treatment. Data are mean ± SD of at least three independent experiments. *p < 0.05, **p < 0.01, ***p < 0.001 as indicated groups. NS, not significant.

Next we investigated whether the inflammatory microenvironment induced by corticotomy influences macrophage polarization. BMDMs were incubated with fresh tissue suspension from mouse palatal gingiva. Real-time PCR showed that levels of M1-phenotype markers TNF-α or IL-1β were significantly higher in the CO + TM group than in the TM group; the levels increased from day 5 to day 14 and then declined on day 21 (Fig. 5A,B). Similarly, levels of M2-phenotype markers Arg-1 or CD206 were significantly higher in the CO + TM group than in the TM group; the levels in both groups increased initially, declined slightly until day 14, and then increased again (Fig. 5C,D).

Western blotting showed that p65 levels in BMDMs increased after incubation with tissue suspension from the CO + TM group between days 5 and 7, after which they began to decrease (Fig. 5E,F). Levels of phosphorylated STAT3 increased on day 5 and declined until day 21, after which they increased again (Fig. 5G,H).

## Discussion

The present work confirms that macrophages are actively involved in corticotomy-accelerated OTM. Briefly, corticotomy of the palatal alveolar bone altered local microenvironment and stimulated macrophage infiltration and polarization. Monocyte/macrophage depletion, which blocked macrophage accumulation and expression, decreased OTM distance. Moreover, the acceleration was significantly impaired when macrophages were depleted, hence confirming that macrophages are at least one of the key players that mediate corticotmy-accelerated OTM.

As an important inflammatory cell type, macrophages contribute to the pathogenesis of multiple diseases by orchestrating the inflammatory response^23,24^. However, no study has ever shown that these cells can be mobilized during corticotomy-accelerated OTM progression. For the first time, we found that there was a strong macrophage infiltration and phenotypic switch at the gingival tissue after supraperiosteal corticotomy on the palatal side. Typically, resident macrophages are well adapted to the steady-state niches^25^; thus unusual challenges such as injuries may mainly attract the infiltration of vascular monocyte-derived macrophage^26^. The gingiva is highly vascular, which explains why macrophage infiltration starts there. With a histologically inseparable proximity to the periodontal ligament (PDL), macrophages in the gingiva can quickly migrate to the PDL, thus providing an important avenue for macrophage infiltration in corticotomy-accelerated OTM.

In addition to increased macrophage infiltration, the microenvironment produced by the corticotomy also triggers dynamic changes in macrophage polarization that are critical for bone homeostasis. We found that there was a significantly increased macrophage polarization toward an M1-like phenotype 5 days after the corticotomy, concomitant with an increased expression of IL-1β and TNF-α, important cytokines for bone resorption^27,28^. Our subsequent TRAP staining confirmed these changes resulted in an activation of osteoclast differentiation, which are consistent with the findings in OTM without corticotomy^15^. Furthermore, M1-like macrophages predominated in the OTM bone microenvironment about three weeks after the corticotmy, when the average tooth movement rate was significantly higher in the CO + TM group on day 21. We believe that the initial increase of average tooth movement rate in the CO + TM group on day 5 may be at least partly due to decreased bone density. Whereas the lag between corticotomy and tooth movement further ascertained that corticotomy-accelerated OTM is not through structurally weakening of the bone, rather it is through a cascade of biological reactions involving macrophages. In comparison, M2-like macrophages did not significantly increase until a later stage of OTM (between days 21 and 28). As production of M2-phenotype markers such as Arg-1 can promote bone formation, this switch may be important for the cessation of bone resorption and initiation of tissue repair^29,30^. However, whether macrophage polarization directly influences OTM is unclear. Further work should focus on the depletion of M2-like macrophage to confirm that the accumulation of M1-like macrophages contributes to OTM under force application.

Many studies have analyzed macrophage responses to LPS signaling through nuclear factor-κB (NF-κB), but this results in ‘activated’ macrophages that are mainly involved in inflammation responses and are likely to be anti-tumoral^31^. In contrast, in their trophic and immunosuppressive functions, M2-like macrophages are shaped by IL-10 and IL-4 or IL-13 that signal to STAT3^32^. Consistent with the previous studies, our data suggest that macrophage polarization toward an M1 phenotype is through activating the NF-κB pathway, while polarization toward an M2 phenotype is through activating the STAT3 signaling pathway.

In summary, revisiting the two specific questions we asked, we ascertain that corticotomy-induced acceleration of OTM is at least partly through stimulating macrophage infiltration and regulating their polarization into M1 and M2 phenotypes, at early and late phases of OTM, respectively. We also reveal that these regulations may be via NF-κB and JAK/STAT3 pathways, respectively.

## Electronic supplementary material

Supplementary Information
