# Supplementary material for: Macrophages mediate corticotomy-accelerated orthodontic tooth movement
Source: Sci Rep. 2018 Nov 14;8:16788. doi: 10.1038/s41598-018-34907-5 (PMC6235963; doi:10.1038/s41598-018-34907-5)
Supplement: Supplementary file 1 — Supplementary Information [file 41598_2018_34907_MOESM1_ESM.docx]

**Macrophages mediate corticotomy-accelerated orthodontic tooth movement**

Yan Wang^1,2,5*^, Hanwen Zhang^3*^, Wen Sun^1^, Siyu Wang^1,2^, Shuting Zhang^1,2^, Linlin Zhu^1,2^, Yali Chen^1,2^, Lizhe Xie^1^, Zongyang Sun^4^, and Bin Yan^1,2^

**Appendix：**3 Appendix Figures and 2 Appendix Tables are available.

**Appendix Figures & Appendix Figure legends**

**
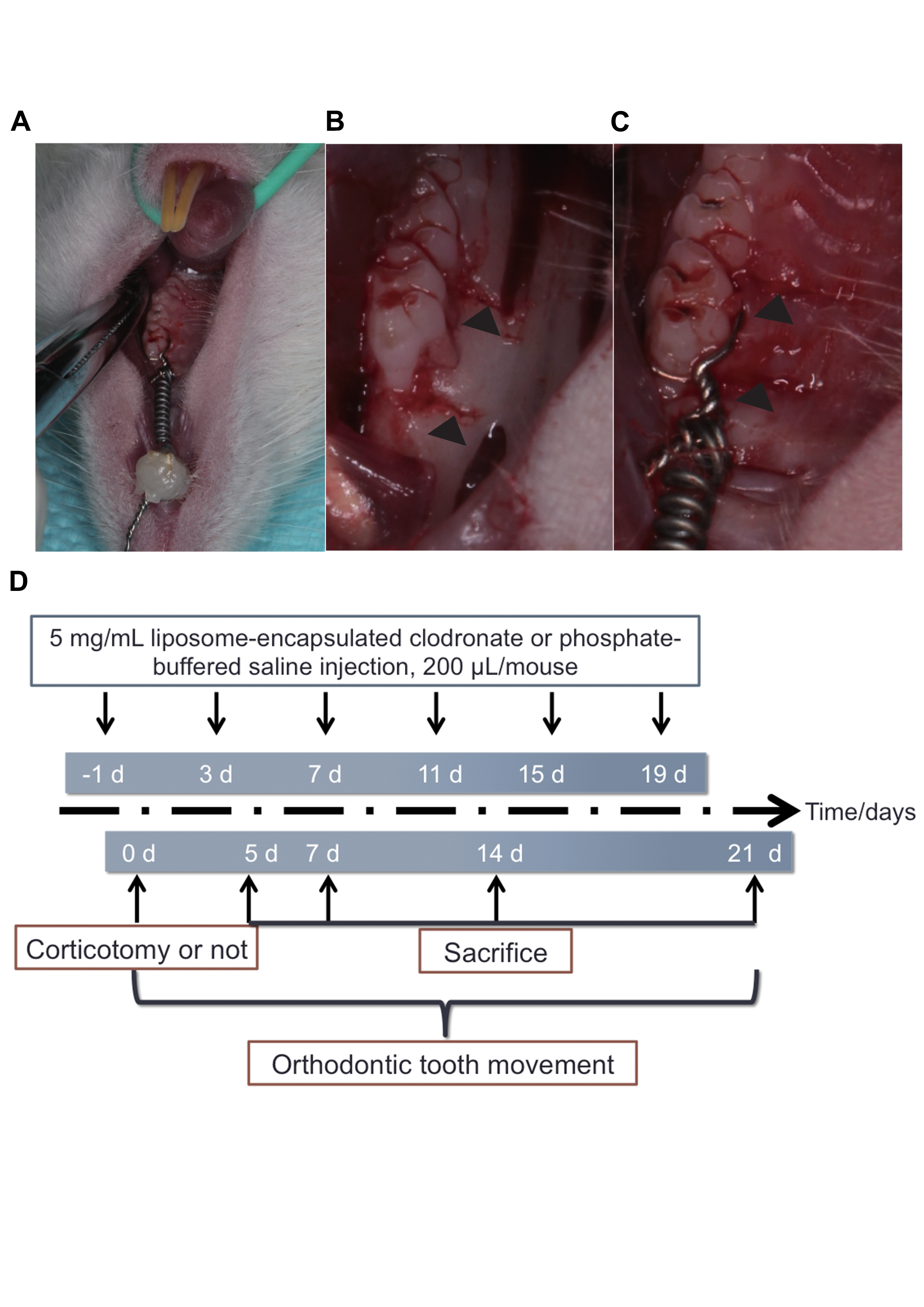
**

Appendix Figure 1. Establishment of a murine model of corticotomy-accelerated OTM and the study design. (A) The left maxillary first molars were ligated to the maxillary incisors using NiTi coil springs, with a force of approximately 60 g for rats. The right maxillary first molar without OTM served as the control. (B) Two vertical corticotomies, 7 mm long and 1 mm deep, were made supraperiosteally to the mesial and distal side of the left maxillary first molar palatal alveolar process in rats using a #11 surgical blade. Arrows represent the position of corticotomies. (C) Intraoral photograph of a rat after corticotomy. The corticotomies were made to the mesial and distal side of the left maxillary first molar palatal alveolar process. Arrows represent the position of corticotomies. (D) For depletion of monocytes/macrophages, mice in both TM and CO + TM groups were randomly divided into two subgroups (n=5-6 per subgroup): one received intravenous injection of liposome-encapsulated clodronate (200 μL/mouse, 5 mg/mL; Liposoma B.V., Netherlands) to deplete monocytes/macrophages; the other received intravenous injection of phosphate-buffered saline (PBS, control). All mice received injections every 4 days starting 1 day before force application until 21 days after force application.


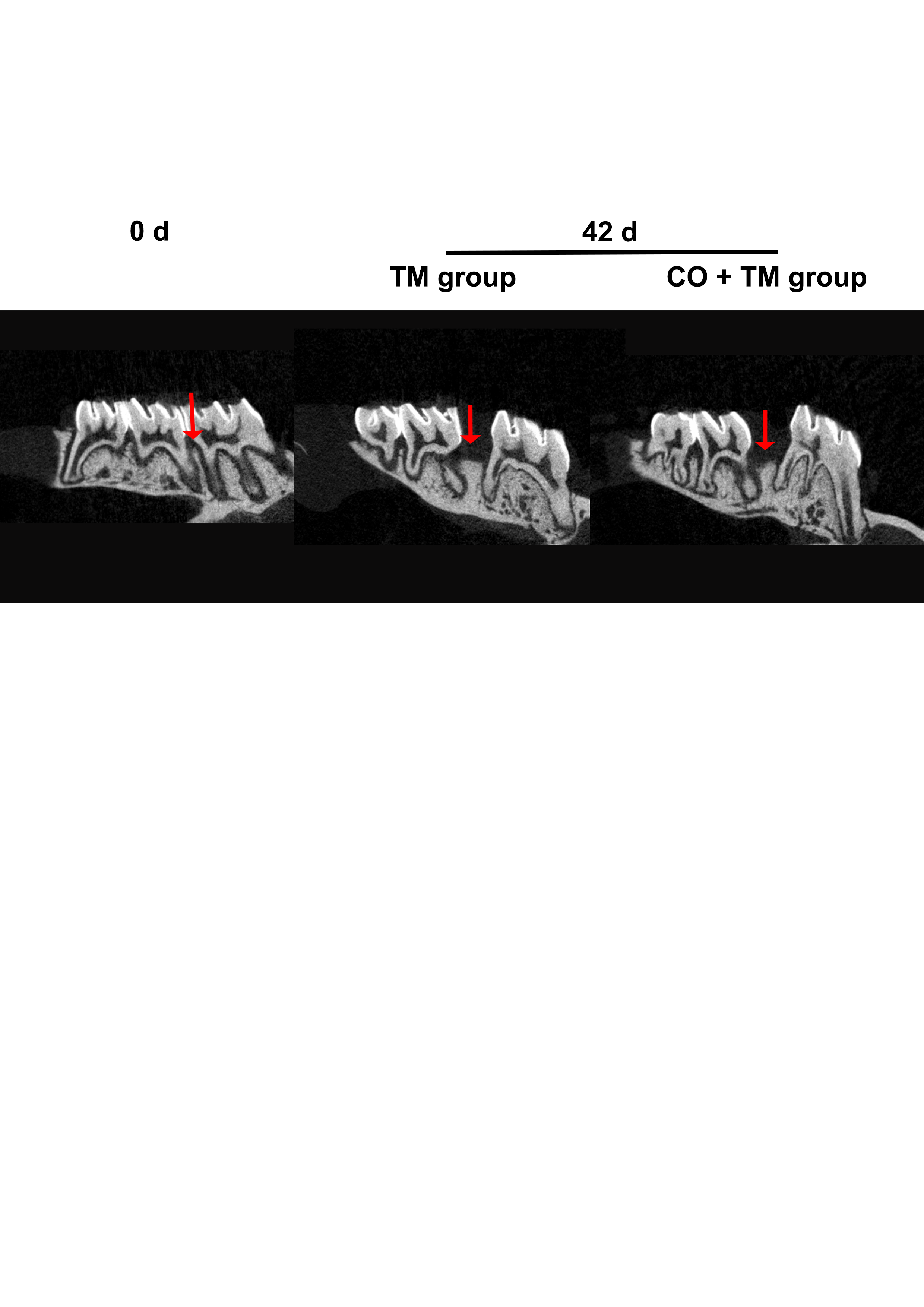


Appendix Figure 2. Representative microcomputed tomography images taken through the centers of the upper left first and second molars in sagittal. Arrows represent the height of the alveolar bone crest at the distal bone ridge of the upper left first molar. (n = 5 or 6).


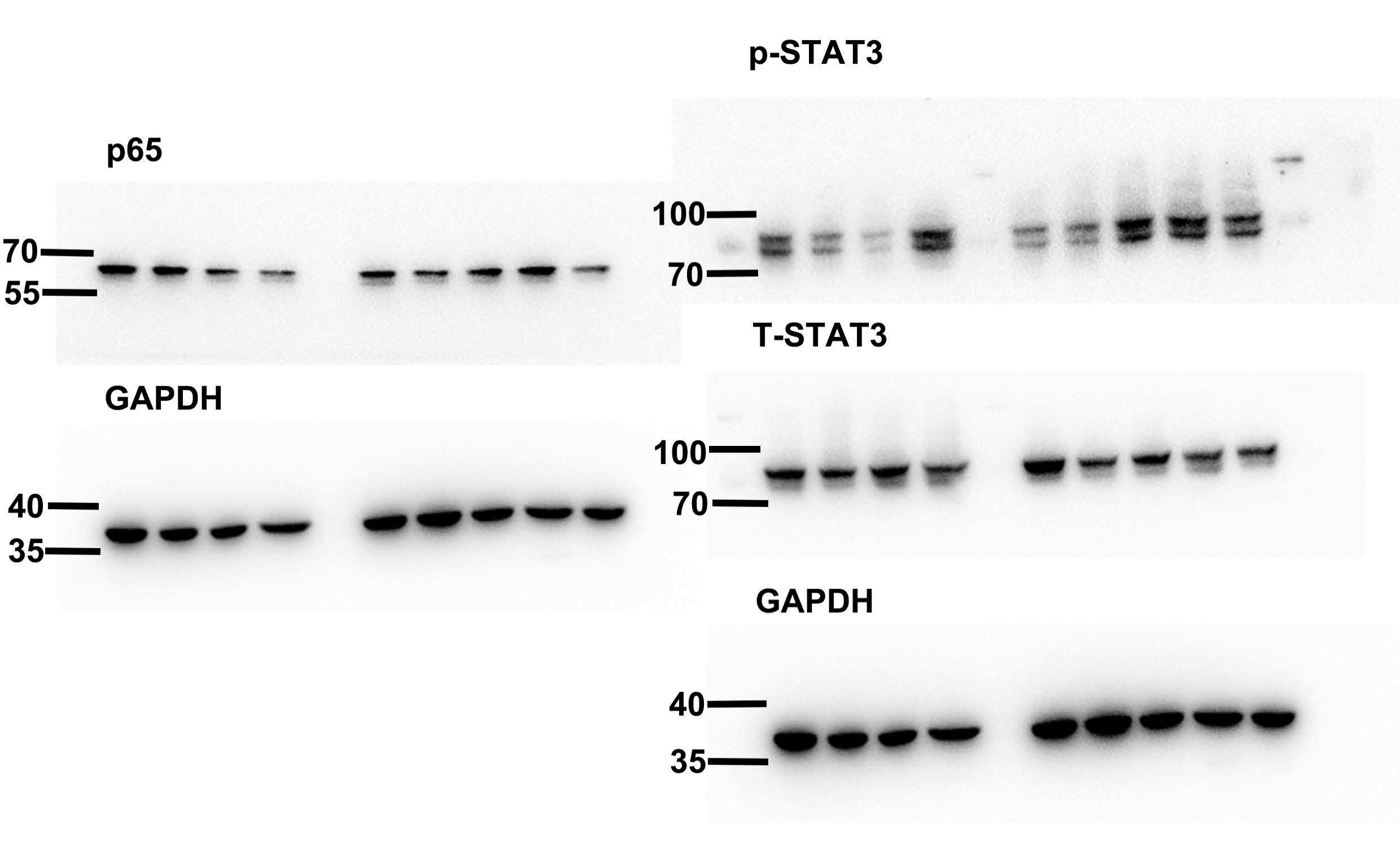


Appendix Figure 3. Full immunoblots for main figure 5.

| **Gene** | **Species** | Sequence |
| --- | --- | --- |
| CD68 | Rat | Forward: 5’-AAACAGTCCAGGCTTCTCCA-3’ |
|  |  | Reverse: 5’-ACATGGCTGGGAACCATTAG-3’ |
| CD11b | Rat | Forward: 5’-GTGAAACCCGAGTGGTTGTT-3’ |
|  |  | Reverse: 5’-TGTGGAGGGACGGTAGTAGC-3’ |
| CD86 | Rat | Forward: 5’-G GACACCCACGGGATCAATTA-3’ |
|  |  | Reverse: 5’-G GCCTCCTCTATTTCAGGTTCAC-3’ |
| CD163 | Rat | Forward: 5’-G TCCGGTTGAAGTTTTGTGACC-3’ |
|  |  | Reverse: 5’-G GTGGTCCCGATGACCGTATT-3’ |
| TNF-α | Rat | Forward: 5’-GGGCTCAGAATTTCCAACAA-3’ |
|  |  | Reverse: 5’-ATCCACTCAGGCATCGACAT-3’ |
| IL-1β | Rat | Forward: 5’-GCAATGGTCGGGACATAGTT-3’ |
|  |  | Reverse: 5’-GACTTGGCAGAGGACAAAGG-3’ |
| Arg-1 | Rat | Forward: 5’-TTGGGAAGACACCAGAGGAG-3’ |
|  |  | Reverse: 5’-GGTTCTGTTCGGTTTGCTGT-3’ |
| CD206 | Rat | Forward: 5’-CAAACCACACATGCACAAAT-3’ |
|  |  | Reverse: 5’-ACCGAAACGTCCCTTTGTTT-3’ |
| GAPDH | Rat | Forward: 5’-GATTTGGCCGTATCGGAC-3’ |
|  |  | Reverse: 5’-GAAGACGCCAGTAGACTC-3’ |
| TNF-α | Mouse | Forward: 5’-CCTCCTCTTTTGCTTATGTT-3’ |
|  |  | Reverse: 5’-CAATTACAGTCACGGCTC-3’ |
| IL-1β | Mouse | Forward: 5’-ACGGACCCCAAAAGATGAAG-3’ |
|  |  | Reverse: 5’-TTCTCCACAGCCACAATGAG-3’ |
| Arg-1 | Mouse | Forward: 5’-GCTCAGGTGAATCGGCCTTTT-3’ |
|  |  | Reverse: 5’-TGGCTTGCGAGACGTAGAC-3’ |
| CD206 | Mouse | Forward: 5’-CTCTGTTCAGCTATTGGACGC-3’ |
|  |  | Reverse: 5’-CGGAATTTCTGGGATTCAGCTTC-3’ |
| GAPDH | Mouse | Forward: 5’-AGAACATCATCCCTGCATCC-3’ |
|  |  | Reverse: 5’-TCCACCACCCTGTTGCTGTA-3’ |

**Appendix Table 1.** Primers used in quantitative real-time PCR

|  | **Antibody** | **Class** | **Clone number** | **Brand** | **Catalog** |
| --- | --- | --- | --- | --- | --- |
| **Flow cytometry** | APC Mouse Anti-Rat CD11b | mAb | WT.5  (RUO) | BD Biosciences | 562102 |
|  | FITC Mouse anti Rat CD86 | mAb | 24F | AbDSerotec | MCA2874T |
|  | FITC Mouse anti Rat CD163 | mAb | ED2 | AbDSerote | MCA342GA |
|  | F4/80 Monoclonal Antibody, PerCP-Cyanine5.5 | mAb | BM8 | ebioscience | 45-4801-82 |
|  | CD11b Monoclonal Antibody, APC | mAb | M1/70 | ebioscience | 17-0112-82 |
|  | CD86 (B7-2) Monoclonal Antibody, FITC | mAb | GL1 | ebioscience | 11-0862-82 |
|  | CD206 (MMR) Monoclonal Antibody, PE | mAb | MR6F3 | ebioscience | 12-2061-82 |
| **Immunohistochemistry** | Anti-CD68 | poly-clonal Ab |  | Abcam | ab125212 |
|  | Anti-CD11b | mAb | EPR1344 | Abcam | ab133357 |
|  | Anti-CD86 | mAb | BU63 | Abcam | ab213044 |
|  | Anti-CD163 | mAb | EPR19518 | Abcam | ab182422 |
| **Western blotting** | p65; RELA Antibody | poly-clonal Ab |  | Proteintech | 10745-1-AP |
|  | Stat3 Mouse mAb | mAb | 124H6 | Cell Signaling Technology | 9139 |
|  | Phospho-Stat3 (Tyr705) XP® Rabbit mAb | mAb | D3A7 | Cell Signaling Technology | 9145 |
|  | Mouse Anti-glyceraldehyde-3-phosphate Dehydrogenase (GAPDH) | mAb |  | Kangcheng Biological | KC-5G4 |

**Appendix Table 2. Primary antibody source utilized in flow cytometry, immunohistochemistry and western blotting (WB).**
